# Supplementary material for: Encapsulated Essential Oils Improve the Growth Performance of Meat Ducks by Enhancing Intestinal Morphology, Barrier Function, Antioxidant Capacity and the Cecal Microbiota
Source: Antioxidants (Basel). 2023 Jan 22;12(2):253. doi: 10.3390/antiox12020253 (PMC9952412; doi:10.3390/antiox12020253)
Supplement: Supplementary file 1 [file antioxidants-12-00253-s001.zip › supplementary Table S2.pdf]

Table S2. The significant differences of functional abundances of KEGG_pathway between the four groups.

Total	KEGG_pathway	Mean.In.KSS	Mean.In.JC	Mean.In.JYG	Mean.In.JYD	Pvalue	FDR
1	ko00623:Toluene degradation	3778.1956	4638.763683	11675.1663	9900.474533	0.00019	0.00147
2	ko00020:Citrate cycle (TCA cycle)	15788.65418	17668.5118	32181.8799	29977.30488	0.00029	0.00147
3	ko00740:Riboflavin metabolism	13103.97027	14926.95415	27255.9201	24955.5311	0.0003	0.00147
4	ko00440:Phosphonate and phosphinate metabolism	2439.7575	2912.686067	4328.05798	4816.079167	0.00031	0.00147
5	ko00960:Tropane, piperidine and pyridine alkaloid biosynthesis	6707.903333	7827.001533	13368.8476	12200.60973	0.00031	0.00147
6	ko00720:Carbon fixation pathways in prokaryotes	16999.87747	19350.47615	35983.8149	33170.05367	0.00034	0.00147
7	ko00480:Glutathione metabolism	5204.66395	7155.345433	12922.4849	11137.51297	0.00034	0.00147
8	ko00360:Phenylalanine metabolism	4679.896167	5917.947683	10116.3821	9084.964933	0.00035	0.00147
9	ko00511:Other glycan degradation	24324.1273	20097.41585	65344.2542	73019.63167	0.00036	0.00147
10	ko04146:Peroxisome	2802.191933	3745.462967	7686.4941	7343.801483	0.00036	0.00147
11	ko03070:Bacterial secretion system	12841.8441	14786.55052	23500.6801	21599.29563	0.00037	0.00147
12	ko00540:Lipopolysaccharide biosynthesis	6248.35835	5724.790683	39868.9145	33131.70288	0.00038	0.00147
13	ko00450:Selenocompound metabolism	17824.68988	19839.30278	30986.2083	29047.23578	0.00039	0.00147
14	ko00600:Sphingolipid metabolism	7577.359833	6928.734167	15175.9284	17015.00108	0.00039	0.00147
15	ko04210:Apoptosis	78.11633333	142.4304333	1831.76948	1770.880917	0.00039	0.00147
16	ko03008:Ribosome biogenesis in eukaryotes	940.60785	1091.856883	1547.76673	1433.508033	0.0004	0.00147
17	ko00770:Pantothenate and CoA biosynthesis	29374.86333	32613.6911	50072.7231	47439.9868	0.0004	0.00147
18	ko03060:Protein export	23450.94675	26760.44058	41526.5711	38755.01083	0.0004	0.00147
19	ko00140:Steroid hormone biosynthesis	597.2109333	615.1496333	1622.5689	1906.254817	0.00041	0.00147
20	ko00430:Taurine and hypotaurine metabolism	9525.53375	11028.0152	20432.5575	18502.68602	0.00041	0.00147

21	ko00660:C5-Branched dibasic acid metabolism	28553.62165	32456.62998	45771.4178	42505.09722	0.00041	0.00147
22	ko04112:Cell cycle - Caulobacter	23487.64042	26104.4577	42114.9728	39396.52217	0.00042	0.00147
23	ko00531:Glycosaminoglycan degradation	15157.85807	10860.97382	25644.0548	30254.2688	0.00043	0.00147
24	ko01053:Biosynthesis of siderophore group nonribosomal peptides	802.45425	986.8791833	2015.87323	2083.1678	0.00043	0.00147
25	ko00130:Ubiquinone and other terpenoid-quinone biosynthesis	6313.28275	6830.00075	16244.946	14771.14425	0.00043	0.00147
26	ko00750:Vitamin B6 metabolism	14734.53252	17074.56853	34697.888	32715.90437	0.00043	0.00147
27	ko04974:Protein digestion and absorption	122.3648	193.3954	2317.7026	2295.004667	0.00043	0.00147
28	ko00670:One carbon pool by folate	28576.4843	31742.05157	50031.1497	47293.08512	0.00044	0.00147
29	ko00860:Porphyrin and chlorophyll metabolism	11181.79805	12590.64568	18806.8458	16955.59508	0.00045	0.00147
30	ko00630:Glyoxylate and dicarboxylate metabolism	10773.64353	12614.34053	20483.1385	19498.218	0.00045	0.00147
31	ko03030:DNA replication	19740.03207	22241.48705	33898.4325	32273.20583	0.00045	0.00147
32	ko03018:RNA degradation	10768.84242	11770.47848	18479.3076	17603.0491	0.00045	0.00147
33	ko05111:Vibrio cholerae pathogenic cycle	2537.543583	2824.174617	6087.2227	5550.2453	0.00045	0.00147
34	ko00230:Purine metabolism	14248.59205	16348.02023	24050.2764	22779.65097	0.00046	0.00147
35	ko00473:D-Alanine metabolism	31415.84833	34094.32223	43737.6822	41077.70388	0.00046	0.00147
36	ko04141:Protein processing in endoplasmic reticulum	685.1505667	781.8837667	1573.13928	1410.3076	0.00046	0.00147
37	ko00190:Oxidative phosphorylation	7613.415383	8345.050317	14155.3982	13466.72988	0.00047	0.00147
38	ko00920:Sulfur metabolism	14068.05405	15884.05918	25096.3247	24807.0927	0.00047	0.00147
39	ko00290:Valine, leucine and isoleucine biosynthesis	37221.87832	41858.46908	60318.5188	56745.3006	0.00048	0.00147
40	ko00760:Nicotinate and nicotinamide metabolism	17330.21888	20421.23788	31213.8341	29414.53463	0.00048	0.00147
41	ko00510:N-Glycan biosynthesis	286.6896833	465.4384	2277.15917	2036.633483	0.00049	0.00147
42	ko00400:Phenylalanine, tyrosine and tryptophan biosynthesis	20212.09792	21606.62402	34179.0902	32807.50405	0.00049	0.00147
43	ko00260:Glycine, serine and threonine metabolism	17689.46778	20349.50452	30692.2117	29203.43535	0.00049	0.00147
44	ko00790:Folate biosynthesis	17264.19373	19237.59387	39408.0849	37453.97303	0.00049	0.00147
45	ko02030:Bacterial chemotaxis	14439.94102	14691.44358	29395.7153	21518.8405	0.0005	0.00147

46	ko02020:Two-component system	5210.42255	6113.02575	9233.89417	8016.60625	0.0005	0.00147
47	ko00521:Streptomycin biosynthesis	25143.12535	27690.59778	48602.1474	46275.88185	0.00051	0.00147
48	ko01055:Biosynthesis of vancomycin group antibiotics	38833.65667	38423.22167	63036.6917	59251.72167	0.00052	0.00147
49	ko00785:Lipoic acid metabolism	17293.26083	19231.18833	39016.7083	37063.51	0.00053	0.00147
50	ko04626:Plant-pathogen interaction	3297.121867	3654.24125	5403.8454	4805.979017	0.00053	0.00147
51	ko00040:Pentose and glucuronate interconversions	9046.642517	11311.27493	20410.1613	20359.92538	0.00053	0.00147
52	ko00710:Carbon fixation in photosynthetic organisms	24613.0612	27815.4339	42996.5583	40841.30777	0.00054	0.00147
53	ko00520:Amino sugar and nucleotide sugar metabolism	17973.61113	19998.11095	31144.995	30286.639	0.00054	0.00147
54	ko00330:Arginine and proline metabolism	12550.92235	14368.5188	21648.5921	21035.44118	0.00055	0.00147
55	ko00970:Aminoacyl-tRNA biosynthesis	28742.75213	31818.96612	43649.4639	40658.29605	0.00055	0.00147
56	ko00052:Galactose metabolism	17914.33357	19287.76913	29788.3454	31007.69535	0.00056	0.00147
57	ko00730:Thiamine metabolism	26145.08622	28199.93667	43527.7306	42749.20063	0.00056	0.00147
58	ko03430:Mismatch repair	27376.85607	30305.01037	44790.9696	42588.76057	0.00056	0.00147
59	ko00500:Starch and sucrose metabolism	15455.51172	17376.73667	26349.0431	25948.07047	0.00056	0.00147
60	ko00908:Zeatin biosynthesis	9359.77375	10530.04458	21087.9904	20235.92833	0.00056	0.00147
61	ko00240:Pyrimidine metabolism	19078.33518	21656.78448	32074.3189	30133.23888	0.00057	0.00147
62	ko00051:Fructose and mannose metabolism	15856.04407	17803.25107	29409.2662	29548.8784	0.00057	0.00147
63	ko02040:Flagellar assembly	9209.6468	8746.144067	18763.75	13533.84285	0.00057	0.00147
64	ko00910:Nitrogen metabolism	8779.451067	10606.2348	16995.6291	15783.578	0.00058	0.00147
65	ko05120:Epithelial cell signaling in Helicobacter pylori infection	2485.138317	2592.57195	4151.4878	3964.058383	0.00058	0.00147
66	ko00780:Biotin metabolism	23572.562	23490.80033	54337.5073	52060.31567	0.00059	0.00147
67	ko00680:Methane metabolism	7702.263433	8860.2235	14540.4533	13870.794	0.00059	0.00147
68	ko03010:Ribosome	26798.16337	29746.72757	43627.7029	40865.96333	0.00059	0.00147
69	ko03440:Homologous recombination	24274.20285	27284.09095	41364.0878	39405.07283	0.00059	0.00147
70	ko00591:Linoleic acid metabolism	4569.7614	4615.510017	10283.1036	10436.70195	0.0006	0.00147

71	ko00471:D-Glutamine and D-glutamate metabolism	31242.85443	37211.07055	58315.1767	53619.80888	0.00061	0.00147
72	ko00250:Alanine, aspartate and glutamate metabolism	26919.64698	30894.62642	48712.6651	46921.7292	0.00062	0.00147
73	ko00350:Tyrosine metabolism	4479.1814	5784.571617	8150.37668	7440.205933	0.00068	0.00158
74	ko00983:Drug metabolism - other enzymes	19624.58438	23629.5012	36055.7633	34374.1176	0.00068	0.00158
75	ko00900:Terpenoid backbone biosynthesis	22111.71483	25257.3499	34266.6217	32119.06612	0.00071	0.00164
76	ko00624:Polycyclic aromatic hydrocarbon degradation	106.55265	272.53215	33.8509833	0	0.00073	0.00165
77	ko03013:RNA transport	677.1245333	788.26355	1445.63933	1377.8422	0.00077	0.00171
78	ko00650:Butanoate metabolism	10759.75457	13452.86022	18954.3461	16874.82608	0.00079	0.00172
79	ko03420:Nucleotide excision repair	13691.72223	15146.42798	19834.6768	18904.69082	0.00079	0.00172
80	ko03410:Base excision repair	15353.39005	17740.32272	23568.6784	22134.62588	0.0008	0.00172
81	ko00564:Glycerophospholipid metabolism	9554.910217	10943.29705	14868.2403	13939.67287	0.00084	0.00177
82	ko00300:Lysine biosynthesis	28602.706	31568.60383	43223.5735	41624.20408	0.00088	0.00185
83	ko00121:Secondary bile acid biosynthesis	12434.34333	17387.285	30190.8083	32229.04	0.00091	0.00189
84	ko00010:Glycolysis / Gluconeogenesis	19129.89655	22169.0403	29620.5742	28446.60638	0.00099	0.00203
85	ko05150:Staphylococcus aureus infection	700.7923333	931.7529833	236.042267	254.2444333	0.00102	0.00206
86	ko04113:Meiosis - yeast	27.4433	57.27283333	165.808667	91.45303333	0.00109	0.00218
87	ko00906:Carotenoid biosynthesis	850.651	1088.30215	320.244333	278.6082167	0.00114	0.00226
88	ko00120:Primary bile acid biosynthesis	1420.714817	2043.812783	3362.60833	3595.319267	0.00124	0.00243
89	ko00340:Histidine metabolism	21030.84587	24804.1415	33964.5838	32726.38965	0.00129	0.00249
90	ko00550:Peptidoglycan biosynthesis	32440.17053	36390.86597	48810.391	45692.25915	0.00137	0.00261
91	ko00410:beta-Alanine metabolism	7929.7371	9794.3924	13346.4448	12563.54177	0.00139	0.00262
92	ko03040:Spliceosome	0	0	0.75518333	0.001666667	0.0014	0.00262
93	ko03050:Proteasome	105.6321	236.1015333	18.5342333	22.02778333	0.00172	0.00319
94	ko03020:RNA polymerase	18570.79865	20277.45255	27027.2022	26355.75688	0.00182	0.00332
95	ko00472:D-Arginine and D-ornithine metabolism	680.8916667	2253.6	5230.795	4162.108333	0.00193	0.00349

96	ko04122:Sulfur relay system	18120.9035	21447.78288	28364.6133	26136.78135	0.00231	0.00414
97	ko00562:Inositol phosphate metabolism	3243.531	4112.588467	6009.39123	5756.320867	0.00248	0.00439
98	ko00620:Pyruvate metabolism	20212.34253	23441.88387	29846.6411	29029.36543	0.00316	0.00555
99	ko00270:Cysteine and methionine metabolism	22416.99765	24985.38845	32080.1146	30829.41405	0.00322	0.0056
100	ko00522:Biosynthesis of 12-, 14- and 16-membered macrolides	0	3.472216667	0	0	0.00341	0.00581
101	ko04512:ECM-receptor interaction	0	1.047616667	0	0	0.00341	0.00581
102	ko01057:Biosynthesis of type II polyketide products	366	1003.416667	28.25	82.75	0.0037	0.00624
103	ko00311:Penicillin and cephalosporin biosynthesis	685.94785	1155.91215	314.565467	269.7942833	0.00474	0.00786
104	ko04962:Vasopressin-regulated water reabsorption	2.505883333	0.043133333	0.5451	1.1745	0.00475	0.00786
105	ko00030:Pentose phosphate pathway	26423.87733	31096.3228	40150.6742	39401.76835	0.00519	0.00849
106	ko00061:Fatty acid biosynthesis	27759.71065	33010.34245	41897.5709	38084.91975	0.00709	0.01151
107	ko00195:Photosynthesis	7467.3366	8817.225983	13886.3413	8590.8427	0.00832	0.01337
108	ko00930:Caprolactam degradation	1087.5531	2467.15785	479.11165	320.4957167	0.00937	0.01492
109	ko00941:Flavonoid biosynthesis	17.61983333	24.2115	185.742833	63.39583333	0.00999	0.01577
110	ko05146:Amoebiasis	171.46675	206.9519667	114.108917	162.7775667	0.01082	0.01693
111	ko03015:mRNA surveillance pathway	0.15125	1.7253	0.08641667	0.074066667	0.01156	0.01791
112	ko03450:Non-homologous end-joining	244.5055	449.0531667	106.399833	152.1841667	0.01355	0.0208
113	ko00363:Bisphenol degradation	872.14	3801.605567	0	0	0.0167	0.02542
114	ko00640:Propanoate metabolism	12094.88853	15012.3955	17731.3204	16197.42327	0.01932	0.02915
115	ko00625:Chloroalkane and chloroalkene degradation	8025.308217	10020.67373	12045.6611	11834.83655	0.02047	0.03062
116	ko00633:Nitrotoluene degradation	7366.338783	7396.440883	6893.84002	5751.396667	0.02066	0.03063
117	ko00621:Dioxin degradation	3111.386417	3713.921433	1986.09927	2140.08165	0.02091	0.03074
118	ko00791:Atrazine degradation	274.1321333	629.9826	625.55165	121.7633333	0.02187	0.03188
119	ko05322:Systemic lupus erythematosus	0.250283333	0.69695	0	0	0.02429	0.0351
120	ko00280:Valine, leucine and isoleucine degradation	7892.189833	10364.00223	12372.2153	11212.44082	0.02449	0.03511

121	ko00903:Limonene and pinene degradation	3246.5785	5290.42045	2713.33803	1804.83455	0.02618	0.03722
122	ko00053:Ascorbate and aldarate metabolism	4069.31045	5500.956933	6286.08935	5890.50605	0.02947	0.04155
123	ko01056:Biosynthesis of type II polyketide backbone	6.111116667	27.44443333	1.94445	0.222216667	0.03133	0.04381
124	ko00281:Geraniol degradation	1718.12375	3903.335433	5217.04937	4256.638967	0.03266	0.0453
125	ko00965:Betalain biosynthesis	23.62833333	49.43166667	15.25	9.666666667	0.03592	0.04943
126	ko00100:Steroid biosynthesis	1042.9826	778.6552167	347.78165	472.05345	0.03948	0.0539
127	ko00909:Sesquiterpenoid biosynthesis	2.7	11.98333333	1.125	0.033333333	0.04358	0.05903
