# Supplementary material for: Encapsulated Essential Oils Improve the Growth Performance of Meat Ducks by Enhancing Intestinal Morphology, Barrier Function, Antioxidant Capacity and the Cecal Microbiota
Source: Antioxidants (Basel). 2023 Jan 22;12(2):253. doi: 10.3390/antiox12020253 (PMC9952412; doi:10.3390/antiox12020253)
Supplement: Supplementary file 1 [file antioxidants-12-00253-s001.zip › supplemetary Table S1.pdf]

Table S1. The relative abundance of the top 40 families of the gut microbiota composition

**Family**	**KSS**	**JC**	**JYG**	**JYD**
Verrucomicrobiaceae	0.368915	0.242681	0.03338	0.106059
Bacteroidaceae	0.006185	0.018016	0.267565**	0.31506**
Ruminococcaceae	0.131162	0.153957	0.07873	0.083169
Peptostreptococcaceae	0.187982	0.17515	0.013975**	0.022273**
Lachnospiraceae	0.084027	0.088015	0.037761	0.060094
Porphyromonadaceae	0.010195	0.004643	0.062996*	0.083909**
Erysipelotrichaceae	0.044219	0.049622	0.013806*	0.024494
Desulfovibrionaceae	0.001388	0.000771	0.076632**	0.045271**
Rikenellaceae	0.002946	0.006448	0.053387**	0.041045**
No_Rank	0.021562	0.012139	0.060251	0.008622
Succinivibrionaceae	0.000756	0.00182	0.053757**	0.042895*
Acidaminococcaceae	0.000262	0.000432	0.048559**	0.044947**
Corynebacteriaceae	0.02147	0.052399	0.006*	0.008437*
Coriobacteriaceae	0.022272	0.030989	0.00327*	0.006386
Staphylococcaceae	0.018153	0.034752	0.001959*	0.006617
Unassigned	0.006601	0.00907	0.028105	0.015301
Prevotellaceae	0.001635	0.00182	0.030418**	0.014715
Fusobacteriaceae	0.000416	6.17E-05	0.027673*	0.008113
Brevibacteriaceae	0.008853	0.022505	0.00071*	0.00145*
Deferribacteraceae	0.001126	0.001296	0.016721	0.012556
Clostridiales_Incertae_Sedis_XIII	0.009779	0.009517	0.003023*	0.002345*
Bdellovibrionaceae	0.009038	0.009718	0.001897*	0.001681*
Eubacteriaceae	0.002144	0.008006	0.004874	0.007157
Sutterellaceae	9.25E-05	0.00017	0.013173**	0.006386*
Veillonellaceae	0.000201	9.25E-05	0.013358**	0.004951
Microbacteriaceae	0.004535	0.009965	0.000663	0.001018
Enterococcaceae	0.002113	0.002915	0.004227	0.005337
Bifidobacteriaceae	0.00765	0.005183	0.001465	0.000278
Dermabacteraceae	0.004627	0.007759	0.000339	0.000416
Rhodospirillaceae	0.000123	0.000185	0.009379	0.001419
Enterobacteriaceae	0.000648	0.000864	0.002437	0.005861
Aerococcaceae	0.00202	0.003609	0.001234	0.002237
Peptococcaceae_1	0.004211	0.000571	0.000787	0.001604
Lactobacillaceae	0.000432	0.00398	0.002052	0.000154
Campylobacteraceae	0.000386	0	0.005399	0.000617
Dietziaceae	0.002067	0.003455	0.000309	0.00037
Brachyspiraceae	0	4.63E-05	0.005939	0
Carnobacteriaceae	0.001296	0.003455	0.000247	0.000787
Streptococcaceae	0.001342	0.001496	0.000941	0.000956
Clostridiaceae_1	0.001188	0.000925	0.001095	0.001003

*, $P < 0.05$; **, $P < 0.01$
