# Supplementary figures and images for: Encapsulated Essential Oils Improve the Growth Performance of Meat Ducks by Enhancing Intestinal Morphology, Barrier Function, Antioxidant Capacity and the Cecal Microbiota
Source: Antioxidants (Basel). 2023 Jan 22;12(2):253. doi: 10.3390/antiox12020253 (PMC9952412; doi:10.3390/antiox12020253)

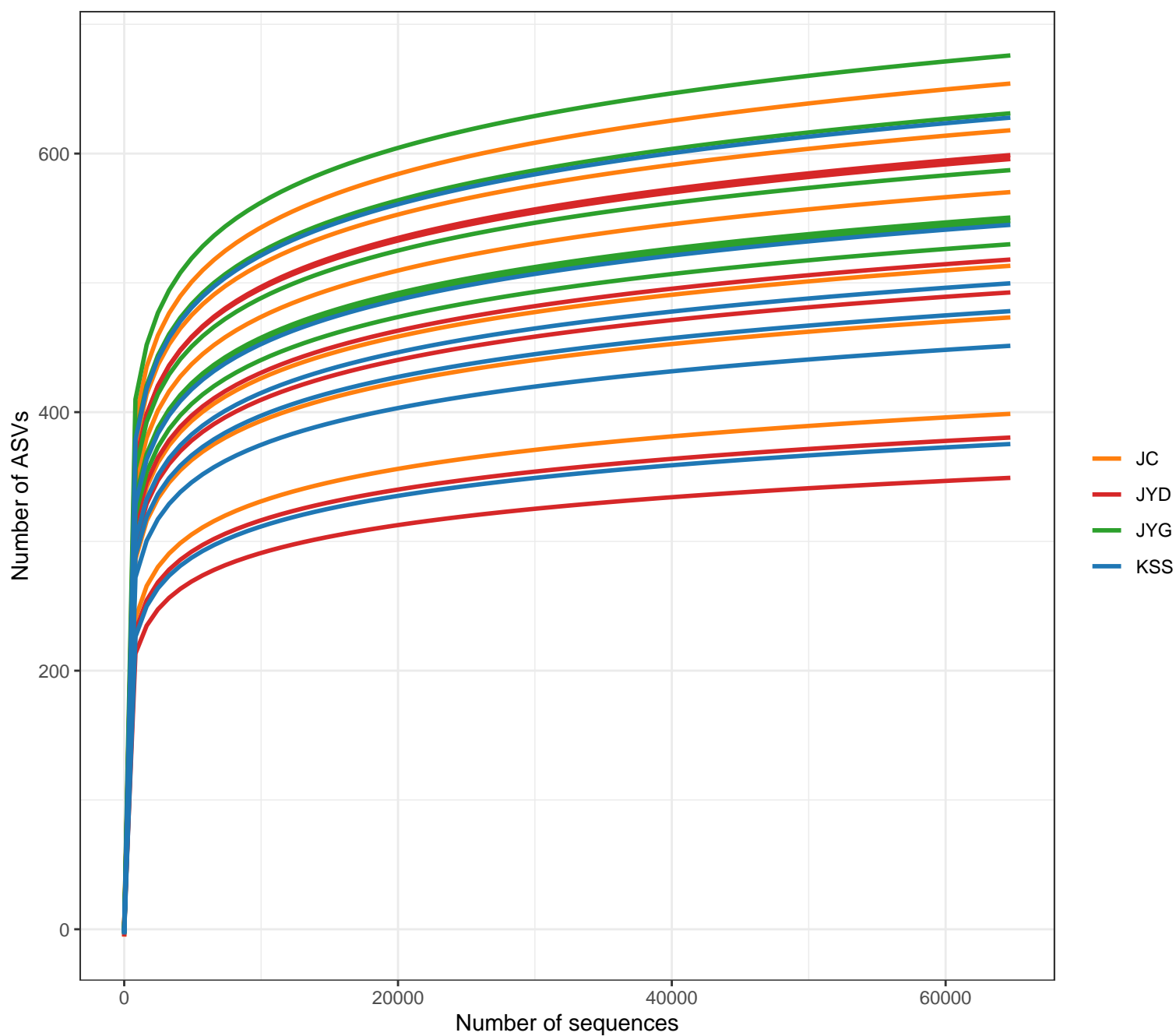

Supplement: Supplementary file 1 [file antioxidants-12-00253-s001.zip › figure S1. The rarefaction curves.pdf]
